# Genetic Syndromes, Maternal Diseases and Antenatal Factors Associated with Autism Spectrum Disorders (ASD)

**DOI:** 10.3389/fnins.2016.00316

**Published:** 2016-07-06

**Authors:** Asher Ornoy, Liza Weinstein- Fudim, Zivanit Ergaz

**Affiliations:** ^1^Laboratory of Teratology, Department of Medical Neurobiology, Hadassah Medical School, Hebrew UniversityJerusalem, Israel; ^2^Department of Neonatology, Hadassah-Hebrew University Medical CenterJerusalem, Israel

**Keywords:** ASD, genetic syndromes, autoimmune diseases, prenatal factors, inflammation, drugs, chemicals, pollution

## Abstract

Autism spectrum disorder (ASD) affecting about 1% of all children is associated, in addition to complex genetic factors, with a variety of prenatal, perinatal, and postnatal etiologies. In addition, ASD is often an important clinical presentation of some well-known genetic syndromes in human. We discuss these syndromes as well as the role of the more important prenatal factors affecting the fetus throughout pregnancy which may also be associated with ASD. Among the genetic disorders we find Fragile X, Rett syndrome, tuberous sclerosis, Timothy syndrome, Phelan–McDermid syndrome, Hamartoma tumor syndrome, Prader-Willi and Angelman syndromes, and a few others. Among the maternal diseases in pregnancy associated with ASD are diabetes mellitus (PGDM and/or GDM), some maternal autoimmune diseases like antiphospholipid syndrome (APLS) with anti-β2GP1 IgG antibodies and thyroid disease with anti-thyroid peroxidase (TPO) antibodies, preeclampsia and some other autoimmune diseases with IgG antibodies that might affect fetal brain development. Other related factors are maternal infections (rubella and CMV with fetal brain injuries, and possibly Influenza with fever), prolonged fever and maternal inflammation, especially with changes in a variety of inflammatory cytokines and antibodies that cross the placenta and affect the fetal brain. Among the drugs are valproic acid, thalidomide, misoprostol, and possibly SSRIs. β2-adrenergic receptor agonists and paracetamol have also lately been associated with increased rate of ASD but the data is too preliminary and inconclusive. Associations were also described with ethanol, cocaine, and possibly heavy metals, heavy smoking, and folic acid deficiency. Recent studies show that heavy exposure to pesticides and air pollution, especially particulate matter < 2.5 and 10 μm in diameter (PM2.5 and PM10) during pregnancy is also associated with ASD. Finally, we have to remember that many of the associations mentioned in this review are only partially proven, and not all are “clean” of different confounding factors. The associations described in this review emphasize again how little we know about the etiology and pathogenesis of ASD. It is obvious that we need more epidemiologic data to establish many of these associations, but if proven, they might be promising avenues for prevention.

## Introduction

ASD is defined by the DSM 5 as a neurobehavioral disorder manifested by persistent deficits in social and communication interaction, deficits in developing, understanding and maintaining relationships, as well as abnormal and fixed interests and repetitive behavior (Kogan et al., 2009; American Psychiatric Association, 2013). Symptoms must be present at early childhood and interfere with daily function. ASD is 4 times more prevalent in males than in females, but in experimental animal models where ASD is induced by neuro-teratogens, there are little gender differences. Mental impairment is common among children with ASD, and a variety of learning and behavioral changes are also prominent in autistic animals. The etiology is diverse, largely unknown, and seems to be the result of genetic and environmental interaction (Kogan et al., 2009; Tchaconas and Adesman, 2013).

Environmental exposures are increasingly being recognized as potential risk factors for ASD, and the possibility that the prenatal and perinatal environment affect fetal programming is an expanding direction for research. Prenatal environment include maternal use of medication, maternal infection and inflammations, and prenatal or perinatal exposure to various substances such as alcohol and heavy air pollution. Of special relevance are prematurity and maternal diabetes.

### Epidemiology

The reported prevalence of autism has increased dramatically over time, from 4 to 5 cases per 10,000 in 1966 to ~1% today (Kogan et al., 2009; Tchaconas and Adesman, 2013). The increase is thought to result increased public awareness, changing diagnostic standards, earlier age of diagnosis, and development of treatment modes. It was also shown that many of the children and adults who were previously diagnosed as having mental retardation meet the DSM 4 and DSM 5 diagnostic criteria of ASD (Shattuck, 2006; Elsabbagh et al., 2012). Some increase, however, may result from “new” environmental causes such as pollution and changing life style.

In the US, for example, the autism and developmental disabilities monitoring network (ADDM network) found in children aged 8 years in 2008 an increase from 1/150 in year 2000 to 1/88 in 2008 and in the 2010 monitoring the rate increased to 1/68 children (Autism Developmental Disabilities Monitoring Network Surveillance Year Principal Centers for Disease Prevention, 2012).

In Israel, judging from the number of children who received childhood disability benefits by the Israeli Insurance Institute because of ASD, the cumulative incidence at 8 years of age at 2011 has increased 10-folds from 1991 and reached 0.49% with a ratio of males/females of about 5 (Raz et al., 2015b).

The more recent global prevalence of autism was estimated to be 0.62% (Autism Developmental Disabilities Monitoring Network Surveillance Year Principal Centers for Disease Prevention, 2012; Elsabbagh et al., 2012). In spite of a wide variation in prevalence between the studies, the authors conclude that there is no evidence of a significant impact of ethnic or socioeconomic factors on the rate of ASD.

It can be presumed that in developed countries the great progress in the diagnosis and treatment of ASD reached a relatively steady state at least in the last 10 years. However, both the incidence and prevalence of ASD continued to rise, implying that some of the increase results from a true increase in ASD rate. For example, in the UK in 1988–92 the incidence was 0.40/10,000 person years and it raised to 2.98/10,000 person years in 2000–2001 (Smeeth et al., 2004) and an in the US an increase was reported in the same states in the US between 2000 and 2008 and a further increase between 2008 and 2010 (Fombonne, 2009; Autism Developmental Disabilities Monitoring Network Surveillance Year Principal Centers for Disease Prevention, 2012). An increased incidence was also reported in Israel (Davidovitch et al., 2013). If this is correct, it may largely result from prenatal environmental causes because genetic, ethnic, socioeconomic, and geographic factors did not change significantly during that time. Recent meta-analysis of seven studies including 1,140,210 children evaluated the association between inter-pregnancy interval and increased risk of ASD, and found OR of 1.9 for interval < 12 months and 1.37 interval >5 years. Proposed mechanisms included folate deficiency, maternal stress, and sustained post-partum inflammation from previous pregnancy for short interval and infertility, unintended pregnancy, and inflammation for long one (Conde-Agudelo et al., 2016). Genetic predisposition was offered by Darbro et al. who found decreased risk of cancer among ASD patients. Despite increased prevalence of rare coding single-nucleotide variations in oncogenes they tend to develop fewer neoplasms. They hypothesized that the protective effect may be due to defects in cellular proliferation and aging (Darbro et al., 2016).

The prenatal causes of ASD can be divided into environmental chemicals (i.e., drugs such as valproic acid, thalidomide, misoprostol; alcohol, cocaine, and toxic metals taken by the mother during pregnancy), exposure to particulate matter air pollution of up to 2.5 micron in diameter (PM2.5), maternal infections during pregnancy (i.e., rubella, CMV), maternal and fetal inflammation (Fox et al., 2012) and maternal diseases (i.e., diabetes mellitus), including autoimmune diseases (Brown et al., 2015) or allergic diseases such as asthma (Croen et al., 2005). In addition, perinatal factors such as perinatal asphyxia, IUGR, RDS, and others were also associated with an increase in the prevalence of ASD (Gardener et al., 2011).

Numerous mechanisms for ASD have been offered, based also on experimental animal models. In addition to complex genetic susceptibility, epigenetic changes have also been proposed (Kogan et al., 2009). Other mechanisms are: immune dysregulation that include abnormal levels of cytokines and growth factors and various fetal and maternal antibodies to brain tissue (Rossignol and Frye, 2012). Additional proposed mechanisms are increased oxidative stress, mitochondrial dysfunction, abnormalities in brain serotonin, abnormal white matter connectivity, decrease number of Purkinje cells in the cerebellum, and neuronal migration defects (Billeci et al., 2012; Grabrucker, 2012; Rossignol and Frye, 2012).

The purpose of the present review is to summarize the data describing genetic syndromes with ASD like behavior and the maternal diseases associated with increased prevalence of ASD. In addition, we summarize the antenatal injurious factors that are known to be related to the etiology and pathogenesis of ASD.

## Genetic syndromes associated with or predisposing to ASD

ASD phenomenology varies between syndromes, but in some it is more common than in the general population (Richards et al., 2015). Several human syndromes derived from a single gene mutation increase the risk for ASD. The more common aberrations are Fragile X syndrome, a mutation in FMR1 (Kaiser-McCaw et al., 1980), Rett syndrome, a mutation in MECP2 (Meloni et al., 2000), tuberous sclerosis, mutations in TSC1 or TSC2 (Green et al., 1994), Timothy syndrome, a mutation in CACNA1C (Gillis et al., 2012), and Hamartoma tumor syndrome, a mutation of the PTEN gene. Copy-number variants that lead to inherited maternal 15q11–13 duplication resulting in Prader-Willi and Angelman syndromes (Ogata et al., 2014) and other duplications like the NPHP1 gene (Yasuda et al., 2014) are also associated with autistic traits. Patients with other syndromes like 22q.11.2 deletion—DiGeorge syndrome (Swillen and McDonald-McGinn, 2015) and neurofibromatosis type 1 (Huijbregts et al., 2015) show behavioral changes that are also common among ASD patients. Multiple other syndromes were associated with ASD, however cases are sporadic (Moss and Howlin, 2009). The development in strategies for the identification of genetic variants led to the description of new syndromic forms of ASD and enabled the association between phenotype and genetic traits.

### Fragile X (FMR1)

Children with Fragile X syndrome which is the most frequent inherited cause of mental retardation have increased rates of ASD. Current estimates suggest that ASD occurs in 21–50% of males with fragile X syndrome, with increased prevalence among individuals with a greater degree of intellectual disability (Moss and Howlin, 2009; Wadell et al., 2013). The Fragile Mental Retardation 1 locus (FMR1) resides in the X chromosome and expansion of triplet repeats in the untranslated region of the FMR1 gene prevents synthesis of the FMR1 gene product FMRP. FMRP is a RNA-binding protein that modulates mRNA trafficking, dendritic maturation and synaptic plasticity. FMRP deficiency leads to dysregulation of other pathways including metabotropic glutamate receptors (mGluR1 and 5), aminobutyric acid A and B pathways, phosphatidylinositol 3-kinase, and mammalian target of rapamycin pathways. FMRP regulates proteins known to be mutated in autism including neuroligins, neurorexins, SHANK, phosphatase, and tensin homolog (Darnell et al., 2011). Deficits in global network organization and decreased clustering with connectome that shift toward randomness were found by Bruno et al among fragile X patients by structural brain network topology evaluation with magnetic resonance imaging (MRI), a finding that was qualitatively similar to what has been described in ASD (Bruno et al., 2016). When 47 children and adolescents with fragile X were assessed for ASD symptoms, most were engaged in self injury behavior, aggression, and stereotypy (Newman et al., 2015). Compared to children with fragile X, those who also had ASD developed slower, had lower developmental level for age that improved over time and performed worse at language tasks (Ballantyne and Nunez, 2016). Boys with fragile X were found to have social smiling less impaired and complex mannerisms more impaired compared to boys with non-syndromic ASD (McDuffie et al., 2015). Besides behavioral Interventions that were proven beneficial, medical therapy based on the pathophysiology of fragile X is currently investigated (Newman et al., 2015). Waddell et al revised the medical treatment modalities based on the up-regulation of the metabotropic glutamate receptor 5 (mGluR5) pathway and down-regulation of the GABA A pathway. The mGluR5 antagonists evaluated in human studies, Fenobam and AFQ056, improved behavior. The GABA B agonist Arbaclofen which is the R-isomer of baclofen and a GABA B agonist that works at a presynaptic receptor improved social behavior of patients with fragile X and ASD. Minocycline, which lowers the levels of matrix metalloproteinase 9 (MMP9), one of a family of proteins important for synaptic plasticity, improved language and behavior among adolescents and young adults with fragile X, however minocycline may cause in young children graying of the permanent teeth when they emerge (Wadell et al., 2013). Human studies with antioxidants like melatonin (Karaaslan and Suzen, 2015; Kwon et al., 2015) and omega 3 fatty acid, vitamin C, vitamin E, and N acetyl cysteine have been tried in single cases. Acetylcarnitine (LAC) was proven beneficial among fragile x patients with ADHD (Wadell et al., 2013).

### Rett syndrome and MECP2 mutations

Rett syndrome, an X linked disease that affects girls, is characterized by neurodevelopmental delay, ASD and seizures. Estimates of rates of ASD in Rett syndrome range from 25 to 40% and up to 97% in individuals with the preserved speech variant of Rett syndrome (Moss and Howlin, 2009). It is caused by mutations in the gene encoding for the methyl-CpG binding protein 2 (MeCP2) that binds to methylated-CpG dinucleotides and influences gene expression. MeCP2 is expressed widely, but is most abundant in neurons of the mature nervous system. MeCP2 duplication syndrome is characterized by autism, intellectual disability, motor dysfunction, anxiety, epilepsy, recurrent respiratory tract infections and early death. The MET receptor tyrosine kinase gene which is a part of the biological network that includes several ASD-associated transcriptional regulators, including FOXP2 and MeCP2 was found reduced in the temporal lobe of subjects with ASD. Regulation of MET transcription by MeCP2 was found *in vitro* in primary human neural progenitors from the olfactory epithelium. Analyses of postmortem temporal cortex samples from Rett syndrome and ASD cases demonstrated sex-based differences in reduced MET expression, with males' perturbation in ASD and females in Rett syndrome (Plummer et al., 2013). In a UK national sample of 91 women with Rett syndrome aged from 4 to 47 years behavioral disturbances included hand stereotypies (99%), low mood/changeable mood (77%), anxiety or inappropriate fear (73%), sleeping difficulties and night-time laughing (64%), teeth grinding (58%), and breath holding (63%) (Cianfaglione et al., 2015). Despite autistic traits Rett syndrome patients have some phenotypic features that differ from ASD patients like increased visual fixation at social stimuli (Schwartzman et al., 2015). Rett patients exhibited a decrease in visual evoked potential (VEP) amplitude that was most striking in the later stages of the disorder and a slower recovery from the peak of the VEP response that was impacted by MeCP2 mutation type (Leblanc et al., 2015). Based on animal studies in which IGF-1 and BDNF supplementation therapies improved neuronal developmental, synaptic maturation and plasticity exerted through the key cell-signaling pathways, PI3K/Akt and MAPK in MeCP2 mutant mice (Castro et al., 2013), a clinical trial in 10 Rett syndrome patients showed improvement in social and cognitive ability with recombinant human Insulin-Like Growth Factor 1 supplementation (Pini et al., 2016).

### Tuberous sclerosis TSC1 or TSC2

Tuberous sclerosis complex is an autosomal dominant disorder caused by mutations in either the TSC1 or TSC2 gene. The syndrome is associated with cerebral cortical tubers and may be complicated by astrocytomas. About 90% suffer from behavioral, intellectual, psychiatric and psychosocial difficulties and up to 60% have the diagnosis of ASD. There is disagreement regarding the association of ASD with the presence of temporal-lobe tubers (Moss and Howlin, 2009). The protein products of TSC1, hamartin and TSC2, tuberin, inhibit Ras homolog enriched in the brain (Rheb) that activates mammalian target of rapamycin (mTOR) complex 1 (mTORC1). Heterozygous defects in either TSC gene prevents the Rheb inhibition and allows for excessive mTOR activation, which induce cell growth and proliferation. Upregulation of the mTOR pathway leads to abnormal dendritic protein synthesis with reduced or dysmorphic dendritic spines and alterations in postsynaptic glutamate receptor-mediated long-term depression, similar to synaptic abnormalities described in ASD. Owing to the presence of cardiac rhabdomyomas being detected by pre/perinatal ultrasonography patients are diagnosed early, allowing prompt detection of ASD symptoms (Davis et al., 2015). In a recent study evaluating 42 patients aged 4–44 years that were diagnosed with Tuberous sclerosis 17 (40%) had ASD that worsened with intellectual disabilities. None of the patients with normal or borderline mental abilities had ASD. The Social Communication Questionnaire (SQL) score was positively associated with epilepsy, seizure onset before age 1 year, infantile spasms and mutations in TSC2, but not tubers localization and gender (Vignoli et al., 2015). The mTOR inhibitors used in treatment of TSC patients for various manifestations were also evaluated for ASD symptoms. Clinical trials with Rapamycin supplementation were inconclusive, some due to the severe intellectual deficits of the evaluated patients. TSC patients with infantile spasms had higher response rates compare to other patients when treated with Vigabatrin which increase GABA availability in the synaptic cleft. It is hypothesized that it may be associated with mTOR suppression found in a murine model (Davis et al., 2015)

### Timothy syndrome—CACNA1C mutation

Timothy syndrome (TS) is a rare dysmorphic disease presenting with cardiac arrhythmias, syndactyly; immune deficiency, intermittent hypoglycemia and neurologic morbidities including seizures, mental retardation, and autism. It is a single nucleotide mutation in the gene encoding the pore-forming subunits of an L-type calcium channel (CaV1.2). The sporadic glycine-to-arginine mutation is located at position 406 in exon 8A (Splawski's terminology). Both variants of TS (the milder TS1 and the more severe TS2) arise from missense mutations in alternatively spliced exons that cause the same G406R replacement in the CaV1.2 L-type calcium channel. Association between non-syndromatic ASD and two SNPs in CACNA1C was found in families of Chinese Han ancestry (Li et al., 2015). Currently there is no specific treatment for ASD in patients with Timothy syndrome. Mexiletine, a voltage-gated sodium channel blocker improved cardiac symptoms in a 2 years old girl; however no data concerning her psychological situation is given (Gao et al., 2013).

### Phelan–McDermid syndrome—*SHANK3* deletion

Deletion of the human *SHANK3* gene near the terminus of chromosome 22q13 is associated with Phelan–McDermid syndrome and autism. Clinical manifestations include dysmorphic face with wide nasal bridge, pointed chin, deep-set eyes, flat mid-face, large ears, long eyelashes, bulbous nose and high-arched palate, hypotonia, developmental delay, and delayed or absent speech. SHANK genes code for large synaptic scaffold proteins of the post-synaptic density. SHANK deletions or mutations were found in about 1% of cases of ASD. Among 760 ASD patients evaluated SHANK1 mutations were found in 0.04% and were present in males with normal IQ and autism, SHANK2 mutations were present in 0.17% of patients with ASD and mild intellectual disability and mutations in SHANK3 were present in 0.69% of the patients with ASD. Those with SHANK3 deletions presented manifestations of the Phelan–McDermid syndrome (Leblond et al., 2014). Using functional MRI to evaluate neural response to communicative vocal sounds, infants with Phelan–McDermid syndrome differed from infants with ASD by their better neural response to communicative vocal sounds in the right superior temporal gyrus (Wang et al., 2016). Children with Phelan–McDermid syndrome were found to have less sensory sensitivity as compared to children with ASD (Mieses et al., 2016).

### Hamartoma tumor syndrome—PTEN mutation

Phosphatase and tensin homolog on chromosome 10 (PTEN) is a tumor suppressor gene that has been reported in autistic individuals with macrocephaly, and also in some syndromes including the Hippocampus Cowdon, the Lhermitte-Duclos disease, and the Bannayan Riley Ruvalcaba Syndrome. More recently, the term PTEN Hamartoma Tumor Syndrome (PHTS) has been used to encompass the range of symptoms identified in PTEN mutation carriers (Leslie and Longy, 2016). McBride et al found in their ASD cohort that 7/99 (7.1%) of the ASD patients and 8/100 (8.0%) of those with mental retardation or developmental delay had PTEN mutations, all of them also had macrocephaly (McBride et al., 2010).

### CNTNAP2 mutations

The CNTNAP2 gene is located in chromosomal region 7q35. A recessive nonsense mutation in the contactin associated protein-like 2 (CNTNAP2) gene was shown to cause a syndromic form of ASD, cortical dysplasia and focal epilepsy syndrome (CDFE). This is a rare disorder resulting in epileptic seizures, language regression, intellectual disability, hyperactivity, and ASD. Neuronal migration defects were found in about half of the patients (Strauss et al., 2006). The CNTNAP2 variant that increases risk for the language impairments in autism was shown to lead to abnormal functional brain connectivity in human subjects (Scott-Van Zeeland et al., 2010). Besides ASD, mutations in the CNTNAP2 gene were also found among patients with Gilles de la Tourette syndrome, ADHD, language disturbances, epilepsy, and schizophrenia (Poot, 2015).

### 15q11-13 deletion or duplication maternal/paternal

Mutations in 15q11-13 are associated with either duplication or gene deletion. Prader-Willi syndrome results from the loss of imprinted genomic material within the paternal 15q11.2-13 locus and deletions, unbalanced translocations, or uniparental maternal disomy. The loss of maternal genomic material at the 15q11.2-13 locus results in Angelman syndrome since normally, only the maternal copy of the Ube3A gene is active in the brain (Lasalle et al., 2015). Region duplications are the most frequent genetic abnormality in autism and the penetrance rate in individuals with a maternally derived duplication is >85% (Cook and Scherer, 2008). Maternally inherited 15q11-13 duplications and triplications are among the most common genomic copy number variants identified in patients with autism. The dosage of an imprinted gene or genes within the duplicated region underlies the autism risk in these patients. E3 ubiquitin-protein ligase, Ube3a is the only gene within the 15q11-13 duplicated segment consistently expressed solely from the maternal allele in mature neurons, and inactivating mutations or deletions of Ube3a cause Angelman syndrome. Treatment attempts to hypermethylate the maternal locus by using pro-methylation oral supplements with folic acid and betaine (Peters et al., 2010) or L-5-methyltetrahydrofolate, vitamin B12, betaine, and creatine (Bird et al., 2011) failed to improve biochemical or behavioral parameters.

### Oxytocin (OXT gene)

Oxytocin is a hormone known for its role in childbirth and lactation. It is synthesized in neurons in the paraventricular and supraoptic nuclei of the hypothalamus and transported via axonal processing to the posterior pituitary, where it is stored in secretory vesicles for release into the portal vascular system to affect target organs, mainly the uterine and mammary glands. Additionally, oxytocin neurons directly project from the hypothalamus to the forebrain, the amygdala, the hippocampus, and the nucleus accumbens. Recent publications showed its role in interpersonal bonding behaviors and psychiatric disorders (Kirsch, 2015). The oxytocin receptor gene is a neuropeptide that contains many single-nucleotide polymorphisms (SNPs) with inconsistency in the effect of these variants on ASD. LoParo et al. revised data from eight studies evaluating 16 SNPs in oxytocin receptor using data from 3941 individuals with ASD from 11 independent samples and found significant associations between ASD and the SNPs rs7632287, rs237887, rs2268491, and rs2254298 (Loparo and Waldman, 2015). Another meta-analysis which also included data bases from Germany found an association with rs237889-A (Kranz et al., 2016). Studies on plasma oxytocin concentrations in children with ASD reported conflicting results, either lower levels (Modahl et al., 1998) or without significant difference to healthy controls (Taurines et al., 2014). Recently Francis et al showed association between OXT rs6084258 and ASD (Francis et al., 2016). Despite no difference between ASD children and controls, higher oxytocin blood levels were found in girls, and were associated with anxiety among the girls and less language impairment in the boys and girls of both groups (Miller et al., 2013). The validity of plasma oxytocin as a potential marker for brain levels was not evaluated among ASD patients, however, blood and CSF levels among non-psychiatric patients undergoing spinal anesthesia for minor procedures failed to show correlation between blood and CSF levels (Kagerbauer et al., 2013).

Oxytocin supplementation can be given either intravenously or intranasal with intranasal being superior due to its being well tolerated and easy to use. Reduction in repetitive behaviors (Hollander et al., 2003) and improved affective speech comprehension (Hollander et al., 2007) were found in ASD adults following oxytocin infusion. Studies evaluating ASD patients showed tasks improvement following a single dose (Aoki et al., 2015). However, some failed to show treatment effects (Althaus et al., 2015). Recent studies used continuous administration in order to evaluate treatment effects on daily life. Continuous intranasal administration over 6-weeks reduced autism core symptoms specific to social reciprocity among adults (Watanabe et al., 2015). Another study that failed to show improvement in social cognition and repetitive behaviors after 6 weeks of treatment found improvements in measures of social cognition and quality of life (Anagnostou et al., 2012). In a double-blind, randomized, placebo-controlled, crossover clinical trial, children with ASD at the age 3–8 years showed global improvement and significant improvement in caregiver-rated social responsiveness following a 5 week course of oxytocin (Yatawara et al., 2015).

The possible role of oxytocin in the etiology of ASD, and its potential use for the improvement of symptoms, is not yet understood and needs more studies.

## Maternal diseases and ASD in the offspring

Diabetes, several autoimmune diseases, infections and inflammatory diseases during pregnancy have been associated with an increased rate of ASD in the offspring. These diseases may apparently occur at any time during pregnancy.

### ASD in offspring of diabetic mothers

Diabetic pregnancies, both pregestational diabetes (PGD) and gestational diabetes (GD) are associated with a large number of pregnancy complications. While PGD may increase the rate of congenital anomalies in the offspring and affect fetal well-being and growth, GD is mainly associated with disturbed fetal growth and increased rate of a variety of pregnancy complications. Both PGD and GD are associated with slight disturbances in postnatal growth and development, also affecting fine and gross motor development and increasing the rate of learning difficulties and Attention Deficit Hyperactivity Disorder (Ornoy et al., 2001). Both also seem to be associated with a slight but significant increase in the rate of ASD in the offspring (Ornoy et al., 2015a).

Most, but not all studies on the possible association of maternal diabetes and ASD show an increased rate, and only recently is the type of diabetes in the mother (T1DM or T2DM or GDM) in relation to ASD in the offspring being considered. Lyall et al. (2012) assessed the possible association of maternal diabetes and ASD in 793 children with ASD from a cohort of 66,445 pregnancies. The highest association was found with maternal GD, as the OR of ASD among children born to mothers with GD was 1.76. This is in slight contrast to other studies which found that the risk for ASD is higher among offspring of mothers with PGD compared to mothers with GDM. Gardener et al. (2009) assessed the possible association of a variety of antenatal maternal factors with ASD in the offspring. Maternal diabetes was among the leading factors associated with ASD, with an odds ratio of 2.07 (95% CI 1.24–3.47). In a very recent study by Li et al. (2016) using the Boston Birth Cohort, both maternal diabetes and pregestational obesity were highly associated with ASD in the offspring. In mothers with PGD and obesity, the hazard ratio (HR) for ASD was 3.91 (95% CI 1.76–8.68) and in obesity and GD the HR was 3.04 (95% CI 1.21–7.63). As cohort and case-control studies are being published, there appears to be a more significant trend toward an association with ASD (Krakowiak et al., 2012). However, there are also studies that could not demonstrate such associations. For example, Hultman et al. (2002) in their case control study on 408 Swedish children with ASD compared to 2040 matched controls, found an association of ASD with a variety of pregnancy associated factors but not with maternal diabetes. Xiang et al. (2015) found in a recent study on pregnant women with preexisting type 2 diabetes. They found an adjusted odds ratio for ASD incidence in the offspring of only 1.21, that was insignificant, but in the offspring of women with GD diagnosed before the 26th week of pregnancy the HR was significant—1.42 (95% CI 1.15–1.74). Guinchat et al. (2012) in their review of 85 studies on the possible association between prenatal, perinatal and neonatal factors and ASD did not find a strong association of maternal diabetes and ASD. In contrast to this review, Xu et al. (2014) culled from 167 publications twelve; three cohort and nine case control studies. For the cohort studies, the pooled risk of maternal diabetes was 1.48 (1.25–1.75, *p* < 0.001), and for the case-control studies, the pooled odds ratio was 1.72 (1.24–2.41, *p* = 0.001). The OR for offspring of mothers with GD was generally lower than for that of mothers with PGD.

In a very recent study looking at the neuropsychiatric morbidity in offspring of 12,642 women with gestational diabetes compared to 218,629 non-diabetic women the investigators found an adjusted odds ratio of 4.4 (95% confidence interval: 1.55–12.69) for ASD. They examined children born during 1991–2014, and excluded prematurity, congenital anomalies, offspring of mothers with pregestational diabetes and other possible etiologic factors (Nahum Sacks et al., 2016).

The mechanism of the association between diabetes and ASD is largely unknown. We should remember that the increased risk found may be related to a variety of pregnancy complications that are common in diabetes, or to effects on fetal growth rather than to complications of hyperglycemia. Judging from the proposed mechanisms of the effects of maternal diabetes on the embryo and fetus, the increase in the rate of ASD in diabetic pregnancies may result from increased fetal oxidative stress, from epigenetic changes in the expression of several genes and it may also be related to the other neurodevelopmental changes induced by maternal diabetes (Ornoy et al., 2015b). It was shown repeatedly that good control of diabetes in pregnancy may reduce the different diabetic complications, but apparently will not completely prevent them. Hence, this is probably also the best way to reduce the diabetic—related ASD. There seem to be no studies relating the prevalence of ASD to the degree of diabetic control.

### Maternal autoimmune diseases

*In utero* exposure to maternal antibodies and cytokines are known potential risk factors for ASD. Recent studies associated familial autoimmune diseases with the development of ASD. Most results come from cohort studies. However, due to the limited number of infants with ASD born to mothers with autoimmune diseases, many studies are small. Three Swedish registries including 1227 children with ASD [childhood autism, Asperger syndrome, or pervasive developmental disorder (PDD)] and 30,693 matched controls (25 controls for each case) evaluated linkage for 19 parental autoimmune disorders and found them weakly associated (maternal OR = 1.6, paternal OR = 1.4). A positive history of rheumatic fever was increased in both parents of ASD children, while type 1 diabetes, idiopathic thrombocytopenic purpura and myasthenia gravis were increased only among mothers (Keil et al., 2010). Out of 689,196 children born in Denmark from 1993 through 2004, a total of 3325 children were diagnosed with ASD. ASD diagnoses included infantile autism (1089 children), atypical autism, Asperger syndrome, and PDD. Children were evaluated for parental autoimmune diseases diagnosed before pregnancy. Increased ASD risk was found in offspring of families with type 1 diabetes, offspring of fathers with endocrine autoimmune diseases and mothers with rheumatoid arthritis and celiac disease. Thyrotoxicosis in the family and the mother, specifically, were related to lower risk of ASD (Atladottir et al., 2009).

Studies on the inter-relation of maternal autoimmune disorders with ASD also showed increased susceptibility to ASD among the offspring. A population-based cohort of 719 children born to 509 mothers with SLE, and a matched control group of 8493 children born to 5824 mothers without SLE found that children born to women with SLE had 1.4% recorded ASD diagnoses compared to 0.6% among the controls (OR 2.25). However, the numbers were small with 10 children of mothers with SLE and 53 children of controls. In addition to maternal SLE, other potential predictors of ASD in the adjusted multivariate analysis were gestational diabetes and male sex (Vinet et al., 2015). The association between maternal autoimmune thyroid disease and childhood autism was evaluated in 1.2 million singleton births with 1132 cases of childhood autism (not including Asperger disorder or PDD) in the Finnish cohort. The cases were matched 1:1 to comparison subjects drawn from the birth cohort who were without ASD. The odds of childhood autism were increased by nearly 80% among offspring of mothers who were positive for anti thyroperoxidase antibodies (TPO-Ab+) during pregnancy, compared to mothers negative for this autoantibody. The association was similar in males and females. There were no associations between maternal clinical or subclinical hypothyroidism or hyperthyroidism, maternal TSH or free T4 and autism (Brown et al., 2015). A follow-up study of 23 mothers with antiphospholipid syndrome (APLS) and their 36 children, and nine SLE mothers with 12 children revealed ASD in 3 children of the APLS mothers. Four children of the APLS mothers, of them the 3 ASD children had persistent anti-β2GP1 IgG antibodies. None of the offspring of the SLE mothers had either neurodevelopmental disorders or anti-β2GP1 IgG antibodies. The ASD cases born to mothers with APLS had normal birth weight, and normal genetic and metabolic work-up. Cerebral MRI performed at the ages of 2.5, 2.6, and 3.4 years were normal in two cases and revealed punctuate hyperintensities in the white matter of one case. None of the children had thrombosis or other antiphospholipid symptomatology (Abisror et al., 2013).

A prospective follow-up of a European multicenter cohort evaluated the long-term outcome and immunological status of children born to mothers with APLS at 3, 9, 24 months and 5 years. Of 134 children, four displayed behavioral abnormalities, which consisted with: autism (1), hyperactive behavior (1), feeding disorder with language delay (1), and axial hypotonicity with psychomotor delay (1). It is noteworthy that the mother of the ASD offspring also suffered from gestational diabetes (Mekinian et al., 2013).

Two large meta-analyses published lately found a significant increase in ASD prevalence among children born to parents with autoimmune diseases. Chen et al. evaluated nine case-control studies and one cohort study published from 1999 to 2015 and comprising of 9775 ASD cases born to mothers with autoimmune diseases and 952,211 controls. Of them, six studies reported a positive association while four did not reveal a substantially significant association. While evaluating the entire cohort they found that children of mothers with autoimmune diseases during pregnancy had a 30% greater risk of ASD. A positive association was identified between maternal autoimmune thyroid disease and ASD in the offspring. No significant difference was found for maternal SLE, inflammatory bowel disease, idiopathic thrombocytopenic purpura (ITP), psoriasis. and rheumatoid arthritis (Chen et al., 2016).

Wu et al evaluated the relationship between family history of autoimmune diseases and risk of ASD in children by a meta-analysis of 11 articles, including 3 cohort studies, 6 case-control studies. and 2 cross-sectional studies published up to Dec 2014. They found in the pooled analysis that family history of all autoimmune diseases combined was associated with a 28% higher risk of autism. Maternal autoimmune diseases were not associated with childhood autism and significant relative risks were only found in case-control studies. Type 1 diabetes in either of the parents, family history of rheumatoid arthritis, hypothyroidism, and psoriasis were significantly associated with higher risk of ASD in children (Wu et al., 2015).

In summary: there is insufficient data to associate maternal autoimmune diseases with increased rate of ASD. However, autoimmune diseases with specific IgG antibodies like TPO-Ab, or anti-β2GP1 that cross the placenta seem to increase significantly the rate of ASD in the offspring

### Pre-eclampsia

Preeclampsia may trigger aberrant neurodevelopment through placental, maternal, and fetal pathophysiologic mechanisms. Preeclampsia results from shallow placentation that may lead to fetal hypoperfusion. Association between pre-eclampsia and ASD was offered when brain MRI of 7–10 years old children (5 boys and 5 girls), offspring of pre-eclamptic pregnancies, revealed enlarged brain regional volumes in the cerebellum, temporal lobe, brain stem, and right and left amygdalae. These offspring also displayed reduced cerebral vessel radii in the occipital and parietal lobes. Enlarged left and right amygdalae were described in ASD and temporal lobe epilepsy (Ratsep et al., 2016).

Recent cohort studies suggested an association between pre-eclampsia and ASD. In the Swedish, population-based, case-control study that included 1216 subjects with autism who were born between 1987 and 2002 and 6080 controls, preeclampsia was associated with 50% increased risk of an autism spectrum disorder (Buchmayer et al., 2009). A recent study from the California Department of Developmental Services that compared 517 ASD cases to 350 controls found that pre-eclampsia complicated the pregnancy of children with ASD more than twice as often as those of controls and the association was more robust in those pregnancies complicated by severe disease (Walker et al., 2015). Two cohort data showed increased prevalence of ASD among pre-eclamptic pregnancies. Analysis of 87,677 births between 1996 and 2002, of the South Carolina Medicaid program, found greater odds of ASD with (OR = 1.69) or without (OR = 1.85) controlling for birth weight (Mann et al., 2010). Analysis of 218,890 singleton live births in Alberta, Canada, between 1998 and 2004 also found greater odds of ASD (OR = 1.49; Burstyn et al., 2010). Compared to these studies there was no association between pre-eclampsia and ASD among 28,967 children born between 1995 and 2008 in Aberdeen city and district (Love et al., 2012). A meta-analysis of 85 studies of pre-, peri,- and neonatal hazards related to PDD, including autism, concluded that there was not enough data in order to determine risk for PDD (Guinchat et al., 2012).

In conclusion: Preeclampsia seems to be an additional risk factor for ASD, but more studies are needed for the conclusive confirmation of such an association.

## The inflammatory and infectious origin of ASD

Adverse intrauterine environment resulting from maternal bacterial and viral infections during pregnancy represent a significant risk factor for several neuropsychiatric disorders including ASD (Adams Waldorf and McAdams, 2013). The association between intrauterine inflammation, infection and ASD is based on both epidemiological studies and case reports.

Many studies associate maternal infection with ASD, and some studies even found that the season or month of birth was significantly related to the risk of ASD. For example, Gardener et al. (2011) found in a meta-analysis that March and August were both suggested as birth months associated with an elevated risk of ASD and hypothesized that a relationship may be caused by seasonal variation in viral or other infections.

### Epidemiological population studies

An increased rate of ASD was found among children born to mothers that were hospitalized due to infection during pregnancy. Atladottir et al. (2010) matched the Danish Medical Birth Register of children born between 1980 and 2005, the Danish National Hospital Register and the cohort data in the Danish Psychiatric Central Register (1,612,342 children with 10,133 cases of ASD). They found that admission to hospital due to maternal viral infection in the first trimester of pregnancy increased the adjusted hazard ratio for ASD in the offspring by 2.98, and due to maternal bacterial infection in the second trimester by 1.42. No specific infectious category was associated with ASD in the offspring. Later, these authors (Atladottir et al., 2012) evaluated in the Danish cohort the association between self-reported maternal common infections and ASD among children born between years 1997–2003, and found that infection with influenza resulted in a hazard ratio of 2.3.

Lee et al. (2015) investigated the Swedish nationwide register-based birth cohort of children born in Sweden in 1984–2007 and followed until December 31, 2011. They found that 3.7% of ASD cases and 2.6% of non-ASD cases had mothers that were hospitalized with a diagnosis of infection during pregnancy. Maternal inpatient diagnosis of infection during pregnancy was associated with increased OR of ASD regardless of whether the infection was bacterial, viral, or other/unknown. The higher risk of ASD was observed during all trimesters: the 1st trimester (OR = 1.24), 2nd trimester (OR = 1.38), and 3rd trimester (OR = 1.36). In a subsample of the total Swedish population, the Stockholm Youth Cohort, the odds ratio for ASD with intellectual disability was 1.50, not related to the type of infection. Contradictory to these findings, Dodds et al. (2011) found that maternal infection during pregnancy did not increase the rate of ASD.

In a meta-analysis of 40 papers published until 2007 Gardener et al. (2009) evaluated the relationships between autism and pregnancy-related factors. Combined together there was no statistical increase in ASD except for maternal rubella with an OR of 1.66. However, when the analysis was limited to the four studies that controlled for multiple covariates or used sibling controls exposure to intra-uterine infections, a significant increase in risk for autism in OR was observed: 1.82 (1.01–3.30).

Case control studies comparing complications of pregnancy including maternal infection have also shown an association between viral maternal infections and ASD (Wilkerson et al., 2002). A similar association was found in women hospitalized during pregnancy due to bacterial infection (Zerbo et al., 2013). There were, however, case control studies that did not find any association of ASD with either maternal viral or bacterial infections (Langridge et al., 2013).

In summary: many but not all population or case control studies have shown a slight to moderate association of maternal infections with ASD. The controversy might be related to the fact that only several specific maternal infections (subgroups of women with infection during pregnancy) are associated with ASD. These are mainly Rubella, CMV and possibly influenza.

### Congenital rubella

The association between Rubella and ASD is based on few population studies and case reports. Berger et al. (2011) calculated that the rubella vaccination prevented an estimated number of congenital rubella cases that ranged from 8300 to 62,250 and that the corresponding ASD prevention estimates ranged from 614 to 4607 cases, depending on the infectious rate and prevalence of ASD among offspring of infected mothers. We should remember that at the time of the rubella epidemics, ASD definition was not the same as nowadays.

The largest study associating congenital rubella with ASD was reported in 1978 by Chess et al. (1978) who evaluated 243 children with congenital rubella, most of them followed until 9 years. Eighteen (7.4%) had autism compared to expected 0.07 (0.35/1000) in the general population at that time. All the children with autism also had other rubella associated anomalies including cardiac, neurologic, hearing and visual problems. Desmond et al. (1978) reported behavioral disturbances among 45% of *29* non-retarded children with congenital rubella at the age of 9–12 years. Carvill et al. (Carvill and Marston, 2002) described 12 young males with congenital Rubella who suffered from sensory imbalance and ASD. Associated morbidities included: visual and hearing impairment and seizures in three. Feldman et al. (1973) evaluated 12 children with autism or autistic traits and compared them with 25 children with a variety of psychiatric diagnoses, 21 children with language delay and 26 normal controls. None had a history of rubella. Sero-positivity for rubella was found to be significantly higher among the children with language delay, 8/21 and among children with autism, 3/12 compared to the other groups and the general population. All the mothers of the sero-positive children were sero-positive for rubella as well. Other reported cases of congenital rubella also described ASD children who suffered from hearing and visual impairment, iris hypoplasia, heart malformations, retinopathy, and seizures (Assumpcao and Kuczynski, 2002; Hwang and Chen, 2010).

### CMV

The diagnosis of maternal CMV is based on medical history, serological studies for CMV or late diagnosis by preserved blood, mostly from Guthrie cards or dried umbilical cord. Koyano et al. (2004) and Sakamoto et al. (2015) used dried umbilical cords for retrospective diagnosis of congenital CMV infection in Japan where obstetric hospitals customarily provide dried umbilical cord to every parent as a symbol of the bond between mother and child.

Yamashita et al. studied seven children with congenital CMV, two of them had ASD. Neuroimaging studies did not vary between those who developed or did not develop autism when seven infants with congenital CMV were evaluated. Three had sub-ependymal cysts and two had calcifications. All had mental retardation (Yamashita et al., 2003).

When children with ASD were assessed, those seropositive for CMV tended to test worse in the major severity scales than the seronegative ones (Gentile et al., 2014). Additionally, Yamazaki et al. found that ASD cases with cochlear implants had lower language and social function than other children with corresponding developmental quotients (Yamazaki et al., 2012). Some studies showed that CMV DNA was detected among ASD cases in a higher prevalence than in the general population; however the numbers were small: 2/27 by Sakamoto et al. compared to incidence of ASD in Nagasaki which was 0.31%, *p* = 0.004 (Sakamoto et al., 2015); 3/76 by Stubbs et al. even though two had multifactorial prenatal causes for autism (Stubbs et al., 1984); 4/26 by Engman et al. with two of them also having cerebral cortical malformations (Engman et al., 2010). Townsend et al. (2013) evaluated 176 infants with congenital CMV born in Sweden and the United Kingdom between 1977 and 1986, and followed them until at least 5 years; none was diagnosed with ASD.

The role of individual placentas in the protection of the fetus against congenital CMV infection is exemplified by the different outcome in association with CMV stigmata, as described by Kitajima (Kitajima et al., 2012) in a triamniotic- trichorionic triplet born at 31 weeks after maternal febrile illness at 24 weeks. Two girls were asymptomatic; the third, a boy, had lower birth weight, increased IGM titer, thrombocytopenia, hepatosplenomegaly, retinitis, brain calcifications, and suffered from ASD. The triamniotic- trichorionic placentas had no fusion or structural abnormalities. Examination of the placenta of the third-born triplet showed that it was paler than the other two and had more severe villitis.

Other reported cases of ASD and congenital CMV also had associated anomalies including brain calcifications and chorioretinitis (Stubbs, 1978; Stubbs et al., 1984; Ivarsson et al., 1990; Sweeten et al., 2004; Lopez-Pison et al., 2005; Ikeda et al., 2006; Kawatani et al., 2010; Dogan et al., 2011).

In summary: There seem to be sufficient studies to associate maternal CMV infection in pregnancy to an increased rate of ASD in the offspring, especially in the children who suffer from other brain manifestations of congenital CMV.

### Influenza

Studies associating maternal influenza and ASD are based on maternal reports. As stated above, Atladottir et al. (2012) found self-reported maternal infection with influenza to be associated with increased risk for infantile autism (adjusted HR: 2.3 [95% CI: 1.0–5.3]). The highest association was found with prolonged episodes of fever.

Zerbo et al. (2013) in a telephone interview, evaluated cases and controls in the Childhood Autism Risk from Genetics and Environment (CHARGE) Study. While comparing data from 538 children with ASD and 421 normal controls in association with maternal influenza during pregnancy, they found that the weighted odds ratio (wOR) between mothers of ASD children and those of children with normal development was 1.26. When they analyzed the association between fever and ASD, the odds ratio for mothers of children with ASD and fever during pregnancy doubled that of mothers of normal controls (wOR = 2.12). They also found that the wOR for ASD was 2.55, for children whose mothers reported fever but did not take anti-pyretic medication. For women who reported fever and took anti-pyretic medications, the wOR for ASD was only 1.30. Hence, if there is an association, it is weak, and apparently due to fever.

### Inflammation

Maternal immune activation and cytokine dysregulation was also shown to be a mediator in the neuro-pathological behavior observed in autism. Maternal immune system and animal models were used to evaluate the role of cytokines in brain dysregulation together with *in-vitro* studies that evaluated the association between inflammatory mediators and neurological components.

#### Studies evaluating maternal immune system

Increased levels of inflammatory mediators obtained from stored samples were found in large population studies. Abdallah et al. (2012, 2013) used a pool of samples from more than 100,000 pregnant women in Denmark and had their biologic samples stored from 1980 to 2004, and matched 421 singleton ASD cases born between 1982 and 2000 for gender and birth year with 820 controls. Levels of Monocyte Chemotactic Protein-1 (MCP-1) which play a role in the maturation of cerebellar Purkinje cells and may serve as a useful marker of abnormal neuronal development, were significantly elevated among ASD cases compared to controls. Using the same cohort amniotic fluid they found that samples from 331 ASD cases compared to 698 controls, had significantly elevated levels of IL-4, IL-10, TNF- α, and TNF- β. Goines et al. (2011) also found Increased levels of IL-4, IL-5, and IFN-gamma in banked serum collected from women at 15–19 weeks of gestation who gave birth to a child ultimately diagnosed with ASD (*n* = 84). Grether et al. (2010) found by examining screening filter paper blood specimens from the California Genetic Disease Screening Program of 213 ASD cases and 265 controls that children with ASD had lower total IgG and that the median value, for Herpes Simplex type 1 Virus IgG was significantly lower compared to controls.

Braunschweig et al. (2008) found reactivity to two protein bands against fetal brain at approximately 73kDa and 37kDa in plasma from 7 of 61 (11.5%) mothers of children with autism. These bands were not found in controls. These children also showed behavioral regression. It is assumed that the presence of specific anti-fetal brain antibodies in the circulation of mothers during pregnancy is a potential trigger to induce a downstream effect on neurodevelopment that may lead to autism. Mazina et al. (2015) investigated the gene-environment interaction by evaluating the interactive effects of maternal infection in pregnancy and the presence of copy number variants which are considered likely to play a contributing role in symptoms of ASD. Participants included 1971 children with ASD between the ages of 4 and 18 years with complete genetic, maternal pregnancy history, and phenotypic information. A statistically significant interactive effect of the presence of copy number variants and maternal infection on autistic symptomatology was observed.

C reactive protein (CRP) is an inflammatory marker that rises in response to infection or inflammation. Analysis of early gestational CRP levels in maternal sera and childhood autism in the Finish birth cohort (consisting of 1.2 million births from 1987–2007) revealed that increased maternal CRP levels were significantly associated with autism in the offspring (OR = 1.12). The increased risk was found in both sexes (Brown et al., 2014). Contradictory to these results, no association between maternal CRP blood levels during the first trimester of pregnancy and ASD in their offspring (children evaluated at 6 years) was found among 4165 pregnant women in Rotterdam that gave birth between 2002 and 2006 (Koks et al., 2016).

#### In conclusion

In children affected by intrauterine infections with rubella or CMV, the association with ASD seems to result from the insult to the nervous system, as all also had other neurological problems as well as visual and hearing impairment, suggesting that ASD is secondary to the primary morbidity. Large population studies did not find a specific maternal infection to be associated with ASD, implying that the association between maternal infection during pregnancy and ASD is apparently related to maternal inflammatory process; hence, maternal immune activation may play a role in neuro-developmental perturbation.

## Exposure to drugs and chemicals during pregnancy and ASD in the offspring

Several drugs and chemicals have been associated with an increased rate of ASD following intrauterine exposure. However, the association was clearly demonstrated only in few drugs. In others, there are either few reports, or the existing literature is contradictory.

### Exposure to SSRI's

Selective serotonin reuptake inhibitors (SSRIs) are among the most used drugs for the treatment of depression especially during pregnancy (Evans et al., 2001; Pereira et al., 2009). They increase extracellular serotonin and are recommended for first-line pharmacological management of depression because they are considered safer and better tolerated than other types of antidepressants. SSRIs and other antidepressant medications cross the placenta and are secreted in breast milk, thus raising concerns about possible adverse effects from fetal and infant exposure. Several studies show a possible association between SSRIs exposure during pregnancy and a higher risk of ASD in children (Croen et al., 2011b; Rai et al., 2013; Gidaya et al., 2014; Harrington et al., 2014). Three out of six recently published case–control studies reported a significantly positive association between SSRI exposure during pregnancy and ASD in children. Odds ratio in the different studies ranged from 2.2 (Croen et al., 2011b) with the strongest effect associated with treatment during the first trimester of pregnancy (OR, 3.8)—to 2.34 (Rai et al., 2013) and the highest was 2.91(Harrington et al., 2014). They found that the strongest association was following exposure in the first trimester of pregnancy. In addition, Gidaya et al. (2014) studied in Denmark a group of 5215 children diagnosed with ASD and 52,150 controls, all born between 1997 and 2006. The OR for ASD doubled among the children born to mothers who used SSRIs at any time during pregnancy.

Recently published large population based retrospective study by Boukhris et al. (2016) reported a significant association between maternal use of SSRI's during pregnancy and the risk of ASD in children. The study included 145,456 children born in Quebec, Canada from 1998 to 2009, of which1054 (0.72%) had at least one diagnosis of ASD. The authors reported that use of antidepressants during the second and/or third trimester was associated with the risk of ASD (31 exposed infants; adjusted hazard ratio, 1.87; 95% CI, 1.15–3.04). Stronger association was found if the mother used SSRI's antidepressants (22 exposed infants; adjusted hazard ratio, 2.17; 95% CI, 1.20–3.93) or used more than one class of antidepressants (5 exposed infants; adjusted HR, 4.39; 95% CI, 1.44–13.32). The risk was persistent even after taking into account maternal history of depression (29 exposed infants; adjusted hazard ratio, 1.75; 95% CI, 1.03–2.97). The use of other antidepressants during the first trimester or the year before pregnancy was not associated with the risk of ASD.

In contrast to these studies, Hviid et al. (2013) found no connection between the use of SSRIs during pregnancy and the increased risk for ASD (OR, 1.20; 95%CI, 0.90–1.61). Similarly, Sorensen et al. (2013) found that children who were exposed to SSRIs during pregnancy have indeed a 50% higher risk for ASD compared with unexposed children, but after controlling for important confounding factors such as maternal history of affective disorder and familial risk factors, they did not detect any significant association between maternal use of SSRI's during pregnancy and ASD in the offspring. Both researchers used data from the Danish civil registration system and Danish health registry systems about children who were born in Denmark between 1.1.96 and 31.12.2005 (Hviid) and until 31.12.2006 (Sorensen). The latest cohort study by Malm et al. from Finland (Malm et al., 2016) examined the rate of ASD among the offspring of 15,729 pregnant women exposed to SSRIs during pregnancy compared to 9651 women with psychiatric disease but no antidepressant treatment and 31,394 non-exposed women and without psychiatric disease. They did not find any increase in the rate of ASD among the SSRIs exposed children, as the rates of ASD were similar to the rate observed in the offspring of mothers with a psychiatric disorder but without treatment. The main finding in this study was an increase in the rate of depression in the SSRI exposed children at adolescence.

In summary: It is still unclear whether there is a direct association between the use of SSRIs during pregnancy and increased rate of ASD in the offspring. Alongside studies which show an association with ASD there are also negative studies. Of special importance is the recent negative study by Malm et al. (2016) as it examined the largest population of SSRI exposed children and adequately adjusted for confounders. It seems more reasonable that maternal depression itself, which is expressed by the use of antidepressants, or other causes, might be responsible for the association. Medical treatment during pregnancy of women with depression should apparently continue in spite of these, rather conflicting studies.

### Exposure to paracetamol

Several studies suggested a possible association between maternal use of paracetamol during pregnancy and a higher risk of ASD among the children. Bauer et al. (Bauer and Kriebel, 2013) examined the association between maternal paracetamol usage or early life paracetamol exposure in males and the prevalence of ASD. They reported positive correlations between ASD prevalence and indicators of both prenatal (*r* = 0.80) and very early life (*r* = 0.98) paracetamol exposures. Liew et al. (2015) followed 64,322 children and mothers enrolled in the Danish National Birth Cohort. They reported that maternal use of paracetamol in pregnancy was associated with ASD with hyperkinetic symptoms only (HR = 1.51 95% CI 1.19–1.92), but not with other types of ASD (HR = 1.06 95% CI 0.92–1.24). More than half of pregnant women in the USA and Europe report using paracetamol and it is the most common drug administered to children; as a result it is difficult to connect between these exposure and ASD). It is obvious that the data is insufficient to draw any conclusion from these studies.

### Exposure to β2-adrenergic receptor agonists

Connors et al. (2005) was the first to examine the effects of prenatal overstimulation of the beta2-adrenergic receptor (B2AR), in 37 dizygotic pair of ASD twins who were exposed to terbutaline, a B2AR agonists, commonly prescribed to treat asthma and other pulmonary disorders and to delay preterm labors. They found increased concordance for autism spectrum disorders in dizygotic twins (relative risk = 2.0), with a further increase in the risk for male twins with no other affected siblings (relative risk = 4.4) related to terbutaline exposure overall.

Croen et al. (2011a) examined maternal use of B2AR agonists during pregnancy among 291 children with and 284 without ASD. No evidence linking B2AR exposure during pregnancy with ASD risk was found. However, they reported that exposure to terbutaline only during the third trimester for more than 2 days may be associated with an increased risk of ASD.

A new case control study by Gidaya et al. (2016) investigated the associations between prenatal use of B2AR agonist drugs and the risk for ASD. They studied 5200 ASD diagnosed cases and 52,000 controls from the Denmark's health and population register. The frequency of B2AR agonist drug exposure in pregnancy was 3.7% (190) for cases and 2.9% (1489) in controls. B2AR agonist use during pregnancy was associated with increased risk of ASD, even after adjustment for maternal asthma and other covariates (OR: 1.3, 95% CI: 1.1–1.5). There was no difference regarding the trimesters of pregnancy during exposure.

### Exposure to valproic acid

Antiepileptic drugs are among the most common teratogens prescribed to women of childbearing age, especially as some of them are also used for psychiatric indications and pain management. Prenatal exposure to antiepileptic drugs (AEDs) is associated with an increased risk of major congenital malformations and delayed cognitive development among the offspring, in a dose dependent manner (Meador et al., 2007; Tomson et al., 2011). Valporic acid (VPA), apparently the most teratogenic AED (Werler et al., 2011), was also found to be associated with impaired cognitive outcomes (Meador et al., 2009). Many reports demonstrate that prenatal exposure to VPA is also associated with a high risk for ASD in the prenatally exposed child in addition to other neurodevelopmental disorders, especially in language development (Moore et al., 2000; Williams et al., 2001; Rasalam et al., 2005; Bromley et al., 2008).

Christianson et al. (1994) were apparently the first to point to of a possible association between intrauterine exposure to VPA and ASD, as observed in four children exposed to VPA during pregnancy. All children had developmental delay and one of them had ASD.

Several years later, Williams et al. (2001) examined five children with ASD and found that VPA was used during pregnancy in all five, but in three in combination with another anticonvulsant. Moore et al. (2000) found among 57 children affected by antiepileptic drugs that four had autistic syndrome and two had Asperger syndrome. Five of them (10.8% of 46 exposed to VPA) were exposed to VPA alone or combined with another AED. Rasalam et al. (2005) evaluated the clinical features and frequency of autistic disorder or Asperger syndrome in 260 children exposed to anticonvulsant medication during pregnancy, by using the DSM IV criteria. They found that VPA was the drug most commonly associated with autistic disorder. The prevalence of ASD in children exposed to VPA alone was 8.9 and 11.7%, among children exposed to sodium valproate in combination with other AEDs. Bromley et al. (2008) examined 296 children of epileptic mothers and 336 children born to mothers without epilepsy. Out of the 632 children from both groups 10 have been diagnosed with ASD, 7 of them were exposed to AEDs. Of those seven children, four were exposed to VPA monotherapy (4/64 6.3%). In addition to these prospective studies, several large retrospective studies have also supported the findings that *in utero* VPA exposure may be linked to an increased risk of ASD (Ornoy, 2009).

Christensen et al. (2013) in a much larger Danish population-based study reviewed the National Population Register with prescription data, psychiatric register and birth records. Records were collated for 655,615 eligible children, with 508 exposed to VPA and 2136 to other anticonvulsants. An increased risk of ASD (Hazard Ratio-HR = 2.9, 95% CI, 1.7–4.9) was found in children exposed to VPA during pregnancy. The risk of ASD was elevated compared to non-exposure when VPA was taken in the first trimester of pregnancy or after the first trimester.

Animal models in rodents using different neurobehavioral measures fully support the human data. Exposure of mice and rats to VPA produced autism-like behaviors in the offspring (Schneider and Przewlocki, 2005; Wagner et al., 2006). Maternal exposure to VPA induced developmental delay, lifelong deficits in social behavior as well as motor deficits, anxiety-like behavior and alterations in postnatal growth and development in the offspring (Schneider and Przewlocki, 2005; Kolozsi et al., 2009; Bambini-Junior et al., 2011). As in humans, anatomical alterations such as reduced number of cerebellar Purkinje cells, damage to cranial nerve nuclei have been described (Rodier et al., 1997; Ingram et al., 2000) as well as enhanced synaptic plasticity of the prefrontal cortex (Sui and Chen, 2012).

In summary: VPA seems to be the drug with the highest association to ASD as proven in human and animal models and should therefore not be used as a first-line antiepileptic drug in pregnant women or in those who plan pregnancy. Similarly, it should not be used as a mood stabilizer for the treatment of psychiatric patients.

### Exposure to thalidomide

Thalidomide is a well-known human teratogen that caused multiple birth defects including limb reduction defects, ocular and cardiovascular anomalies. Thalidomide is still used in the treatment of leprosy and multiple myeloma (Ito et al., 2010). Stromland et al. (1994) have reported increased number of incidences of autism in children exposed prenatally to thalidomide. At least four of the 100 thalidomide embryopathy patients met full criteria for DSM III R autistic disorder- much higher prevalence then in the general population. The critical window of exposure to thalidomide was in the first trimester, mainly days 20–35 post fertilization. Because thalidomide is generally used in elder patients, no newer data was published regarding this possible effect, making it difficult to accept or deny this possible association, or to calculate the relative risk.

### Exposure to cocaine

Cocaine use during pregnancy has many deleterious effects on both the mother and the fetus, (Singer et al., 1993; Fox, 1994). Cocaine, which is used nowadays mainly as a recreational drug crosses the placenta and the fetal blood-brain barrier readily, and may affect the fetal central nervous system (Roe et al., 1990). Its use during pregnancy may have a variety of deleterious effects on the fetus including preterm delivery, intrauterine growth retardation, placental abruption, stillbirth, and neonatal death (Singer et al., 1993; Fox, 1994). Several studies reported a possible association between the use of cocaine during pregnancy and the development of ASD in the offspring.

Davis et al. (1992) examined 70 children with a history of prenatal cocaine exposure, in Harlem, New-York and found that nine of the children met the DMS III R criteria for autistic disorder—11.4%, much higher than the national average at that time. Harris et al. (1995) examined three, 25–36 month old children with prenatal exposure to cocaine, alcohol and other illicit drugs. They reported that all three children showed a notable pattern of autistic-like behaviors.

We could not find other studies associating prenatal cocaine exposure with ASD. Hence, judging from the paucity of studies, in spite of relatively high use, there seems to be insufficient data to support such association. Hence, the relative risk could not be calculated.

### Exposure to ethanol

Fetal alcohol syndrome (FAS) is a pattern of physical and neurodevelopmental abnormalities which develop in some of the children who were exposed to high levels of alcohol during pregnancy. It is characterized by prenatal and postnatal growth deficiency, CNS dysfunction including mental retardation and behavioral abnormalities, a distinctive pattern of facial features and major malformations of several organs. Some studies have shown a connection between FAS and an increased risk of ASD.

Nanson (1992) described six children aged 6–15 years, out of a data base of 326 FAS individuals who showed the physical phenotype of FAS and also presented a behavioral phenotype typical of autism spectrum disorder. The children also suffered from significant retardation of their cognitive and social skills. These data is representing an incidence of ASD in 1 of 54 cases of FAS.

Aronson et al. (1997) found that among 24 children born to mothers who used high doses of alcohol during pregnancy, two had Asperger syndrome and one had autistic-like behavior. There was a clear correlation between the occurrence and severity of ASD and the degree of alcohol exposure *in utero*. Landgren et al. (2010) assessed 71 children adopted from Eastern Europe between 4.8 and 10.5 years of age and found fetal alcohol syndrome in 37 of the children (52%) and autism in 6 of the children (9%), suggesting a positive association between prenatal alcohol consumption and ASD.

The association between maternal alcohol consumption in pregnancy and ASD does not seem to exist with low alcohol intake. In a large population-based cohort study Eliasen et al. (2010) evaluated a total of 80,522 mother-child pairs. Almost half of the women reported an average weekly intake of at least half a standard alcoholic drink (45%), and approximately one-quarter of the women reported at least one episode of binge drinking during pregnancy. They found no positive associations between ASD and average alcohol consumption or number of binge drinking episodes during pregnancy Adjusted hazard ratio for alcohol consumption of 0.5–1.5 glasses of wine per week (119 cases) = 0.84, 95% CI: 0.68–1.05; adjusted hazard ratio for single binge drinking episode (51 cases) = 0.72, 95% CI: 0.53–0.97.

In summary: Although the existing data of the association between high amounts of alcohol consumption in pregnancy and the risk for ASD in children (especially those with FAS) is well established, the relation to lower amounts ingested is not yet established.

### Exposure to misoprostol and moebius sequence

Misoprostol is a prostaglandin analog drug widely used to induce medical abortions (Gonzalez et al., 1998). It is also often prescribed for the prevention and treatment of gastric ulcers. Möbius sequence is a rare congenital disorder, characterized by uni-or bilateral eye-face palsy due to damage to cranial nerve nuclei, associated with muscle or skeletal malformations in the upper or lower limbs, with an estimated prevalence of 1:50,000 cases in the general population (Stromland et al., 2002; Verzijl et al., 2003). Among the etiological factors responsible for Möbius sequence, apart from genetic causes, is also intrauterine exposure to teratogens mainly misoprostol (Bandim et al., 2003; Vauzelle et al., 2013). Judging from the literature, it is expected that about 1% of children exposed *in utero* to misoprostol will have Möbius sequence (Gonzalez et al., 1993; Marques-Dias et al., 2003).

Johansson et al. (2001)analyzed 25 Swedish individuals with Möbius sequence. Six patients met all diagnostic DSM III R criteria for autism which is a much higher frequency than that of the general population. These findings suggest a strong association between Möbius sequence and ASD. All these patients also had mental retardation.

Bandim et al. (2003) examined 23 Brazilian patients with the age range of 1–11 years, which had been diagnosed as having Möbius sequence based on clinical findings. They identified seven children with ASD of whom four (57.1%) had a positive history of prenatal exposure to misoprostol during the first trimester of pregnancy. They concluded that the prevalence of ASD found in Möbius patients is around 26.1%, much higher than the general population, thus suggesting a strong association linking the two pathologies.

The correlation between misoprostol exposure during pregnancy and ASD is suggested on the basis of reports published in the literature. However, since most, if not all affected children also had Mobius syndrome, it is difficult to directly associate misoprostol exposure to ASD. We found no reports on an association of ASD to misoprostol exposure in exposed children that do not present the symptoms of Moebius syndrome. It should be remembered that the experimental models of VPA—induced ASD in rats and mice have also caused damage to the cranial nerve nuclei, similar to the damage observed in Mobius syndrome (Rodier et al., 1997; Ingram et al., 2000; Sui and Chen, 2012). Hence, it is possible that the relation of misoprostol—induced Mobius syndrome to ASD is not a result of the direct effects of misoprostol but rather from the damage to the cranial nerve nuclei.

### Folic acid deficiency

Folic acid deficiency is associated with a variety of adverse outcomes in the offspring, especially Neural Tube Defects (NTD). Folic acid deficiency was therefore also proposed as a possible risk factor for ASD.

Schmidt et al. were the first to report that mothers of children with autism were less likely, than those of typically developing children, to report having taken prenatal vitamins, including folic acid, during the 3 months before pregnancy or the first month of pregnancy (OR, 0.62; 95% CI, 0.42–0.93). Later, Schmidt et al. (2012) examined the effect of maternal folic acid intake on the ASD risk in a group of 429 children with ASD and 278 normal children and found that mothers of normal children had a significantly greater mean folic acid intake during the first month of pregnancy compared to mothers of children with ASD. A mean daily folic acid intake of ≥600 μg during the first month of pregnancy was associated with reduced ASD risk (OR, 0.62), and risk estimates decreased with increased folic acid (P-trend = 0.001). The association between folic acid and reduced ASD risk was strongest for mothers and children with MTHFR 677 C > T variant genotypes.

In a prospective Norwegian cohort study, Surén et al. (2013) followed up 85,176 children of whom 270 had been diagnosed with ASD and found a significantly higher rate of Autism in children who were not exposed *in utero* to folic acid (0.21%) compared to children of mothers who took 400 μg or more folic acid (0.10%) during a month before and 2 month after the start of pregnancy (OR, 0.6).

Recently, DeVilbiss et al. (2015) summarized the existing literature regarding folic acid deficiency and ASD and discussed the limitations of the different studies. He concluded that the data associating Folate deficiency with a higher risk of autism is yet inconclusive. Hence, it is obvious that more studies are needed to elucidate the possible association of maternal folic acid deficiency with ASD.

## Exposure to high levels of environmental agents

### Exposure to air pollution

Increased air pollution is one of the more common signs of our modern life, and its possible effects on health are extensively investigated. In spite of great difficulties in measuring the extent of air pollution and the elucidation the large number of possible confounding factors, more and more studies are being published associating heavy air pollution to ASD.

The first study was apparently conducted in San Francisco Bay area in 2006 by Windham et al. (2006). They examined the effects of prenatal exposure to 19 hazardous air pollutants in 284 children with ASD compared to 657 controls. Elevated risk for ASD was found in adjusted analyses of the top quartile of exposure to chlorinated solvents and heavy metals [95% CI, 1.1–2.1]. In this study, however, the exposure was estimated 2 years after birth when ASD diagnosis was established. Hence the effects might be attributed to postnatal effects of these pollutants.

Kalkbrenner et al. (2010) examined prenatal exposure to 35 hazardous air pollutants in 383 children with ASD and 2829 control children with speech and language impairment. They found that prenatal exposure to hazardous air pollutants including methylene chloride (OR, 1.4; 95% CI, 0.7–2.5), quinoline (OR, 1.4; 95% CI, 1.0–2.2), and styrene (OR, 1.8; 95% CI, 1.0–3.1) were associated with a higher risk for ASD.

Volk et al. (2011) investigated the association between ASD and proximity of residence to freeways and major roadways during pregnancy and near the time of delivery, in a group of 304 children with ASD and 259 controls. They found that maternal residence during the third trimester (OR, 2.22; 95% CI, 1.16–4.42) and at the time of delivery (OR, 1.86; 95%CI, 1.04–3.45) were more likely to be near a freeway (≤ 309 m) for the ASD children than for the controls. They also found (Volk et al., 2013) in an additional study that, during gestation, children with ASD were more likely to live at residencies that had the highest quartile of exposure to traffic-related air pollution (OR, 1.98; 95% CI, 1.20–3.31) compared with control children. Regional exposure during pregnancy with nitrogen dioxide and particulate matter < 2.5 and 10 μm in diameter (PM2.5 and PM10) were also associated with autism.

Similarly, Becerra et al. (2013) studied the influence of exposures to traffic-related air pollution during pregnancy on the occurrence of ASD with controls matched by sex, birth year, and gestational age. They found a small but significant 12–15% relative increase, per interquartile range, in odds of autism for ozone (OR, 1.12, 95% CI: 1.06–1.19; per 11.54-ppb increase) and particulate matter ≤ 2.5 μm when mutually adjusting for both pollutants.

Roberts et al. (2013) estimated the association between levels of hazardous air pollutants at the time and place of birth and ASD in 325 children with ASD and 22,101 controls. Prenatal exposures to the highest quintile of air pollutants, such as diesel, lead, manganese, mercury, and methylene chloride, were significantly associated with ASD, with odds ratios ranging from 1.5 (for overall metals measure) to 2.0 (for diesel and mercury). For most pollutants, associations were stronger for boys (279 cases) than for girls (46 cases) suggesting a significant gender difference.

Raz et al. (2015a) examined the association between maternal exposure to particulate matter (PM) air pollution during pregnancy and the risk of ASD compared to controls. They found that PM2.5 exposure during pregnancy was associated with increased risk of ASD, with an adjusted odds ratio for ASD per interquartile range of 1.57 (95% CI: 1.22, 2.03). The association between ASD and PM2.5 was stronger for exposure during the third trimester (OR, 1.42 per inter-quartile range increase in PM2.5, 95% CI, 1.09–1.86).

In a recent, population based case–control study in Pennsylvania, Talbott et al. (2015) found that living in areas with higher ambient levels of styrene and chromium during pregnancy and the first and second years of life is associated with increased risk of ASD with borderline effects for polycyclic aromatic hydrocarbons (PAHs) and methylene chloride.

In contrast to these studies mostly carried out in the United States, showing a consistently positive association between exposure to several air pollutants during pregnancy and diagnosis of ASD, a much larger, recently published analysis of four European population-based cohorts appears to contradict those findings, reporting no associations between ASD and prenatal air pollution exposure (Guxens et al., 2016).

This research compared between nitrogen oxides and various sizes of particulate matter concentrations at the participants' birth home addresses during pregnancy and the presence of autistic traits, but not formal diagnoses of ASD, in more than 8000 children. No associations were found between the presence of autistic traits and prenatal exposure to any of the air pollutants (odds ratio = 0.94; 95% CI: 0.81, 1.10 per each 10-μg/m3 increase in NO2 pregnancy levels). The researchers explained the contradictory findings by the fact that previous case–control studies selected children with a diagnosis of ASD, whereas this study examined children with autistic traits from population-based birth/child cohorts. An alternative explanation could be the possible differences in air pollution levels and sources.

It can therefore be concluded that air pollution might be a contributing factor in the risk for ASD, but the data is as yet inconclusive.

In summary: these case control studies suggest that there is a positive association between maternal exposure to air pollution during pregnancy and ASD in children, especially following exposure in the third trimester. Although small PM may cross the placenta, there might also be other factors in the air pollutants that are associated and were not measured.

### Exposure to pesticides

Few studies have examined the effects of *in utero* exposure to pesticides such as organophosphates and carbamates on the neurobehavioral development and the risk of ASD in children. Each study reported an association with ASD.

Roberts et al. (2007) examined the association between maternal residence near agricultural pesticide applications during key periods of gestation and ASD in children. They reported that ASD risk was higher in children whose mothers lived near agricultural applications of organochlorines during the first trimester of pregnancy (OR 6.1) and decreased with increasing distance from such fields.

Shelton et al. (2014) evaluated whether residential proximity to agricultural areas and therefore increased exposures to pesticides during pregnancy is associated with ASD. They found that residence near organophosphates at some point during gestation was associated with a 60% increased risk for ASD, higher for third-trimester exposures (OR, 2.0), and second-trimester chlorpyrifos applications (OR, 3.3). Also, children of mothers residing near pyrethroid insecticide applications just before conception or during the third trimester were at greater risk for ASD with ORs ranging from 1.7 to 2.0. We can summarize that due to the paucity of studies in spite of extensive use, an association between ASD and pesticides is plausible, but it was not yet established.

### Exposure to heavy metals

Maternal exposure to heavy metals, such as mercury, lead, and cadmium, has been suggested to be associated with neurodevelopmental disorders including mental retardation, inattention, learning difficulties, and possibly ASD (Mendola et al., 2002). Although there is more data on mercury, most available data suggests that maternal exposure to mercury does not play an important role in the development of ASD. Moreover, several of the studies were carried out on the affected children and not on their mothers, hence making the association with pregnancy an unproven possibility (Kern et al., 2007). In that respect we should add that Kern et al. (2007) found that sulfhydryl reactive metals: arsenic, cadmium, and lead were reduced in the hair of 45 children with ASD compared to controls. They suggested that the children with ASD have a problem in secreting these sulfhydryl reactive metals and their accumulation may contribute to the symptoms of ASD.

Geier et al. (2009) examined the association between maternal dental amalgams (containing 50% mercury) and the severity of ASD diagnosis in a group of 100 autistic children. They found that a rise in autism severity correlated with an elevation in the number of dental amalgams in the mother during pregnancy. However, Yau et al. (2014) investigated the association between ASD and levels of total mercury measured in maternal serum from mid-pregnancy and infant blood shortly after birth in 84 children with ASD and 159 control children. They found no significant associations between ASD and mercury levels in maternal serum samples (OR, 0.96; 95% CI, 0.49–1.90) or in newborn blood samples (OR, 1.18; 95% CI, 0.71–1.95).

Van Wijngaarden et al. (2013) examined prenatal exposure to methyl mercury in 1784 children and young adults, by measuring maternal hair samples collected at or near the time of birth. They found no significant association between ASD and prenatal methyl mercury exposure.

It can therefore be concluded that there in insufficient data to associate prenatal exposure to heavy metals, especially mercury, to a higher occurrence of ASD in the offspring.

### Exposure to cigarette smoking

Heavy maternal cigarette smoking during pregnancy is associated with an increased rate of spontaneous abortions, preterm delivery, reduced birth weight, immune system difficulties such as asthma and allergies and a relatively high rate of learning disabilities and attention deficit disorders later in life (Castles et al., 1999; Stene-Larsen et al., 2009; Kiechl-Kohlendorfer et al., 2010). Several studies have examined whether prenatal exposure to heavy tobacco smoke is also associated with ASD, and the findings are inconsistent. Hultman et al. (2002) and Larsson et al. (2009) found a mild association between smoking during pregnancy and the risk of childhood autism (OR, 1.4 and 2.09, respectively).

Kalkbrenner et al. (2012) assessed the association between maternal smoking during pregnancy and ASD among 3315 children with ASD and 630,674 control children at 8 years of age. A slightly positive association (OR 1.26) was found only for “ASD not otherwise specified” (ASD-NOS), which disappeared after correcting for possible confounding factors. Similarly Tran et al. (2013) examined 4019 ASD cases and 16, 123 controls, in a nested case-control study based on the Finnish Prenatal Study of Autism (FIPS-A) and found no connection between maternal smoking and childhood autism or Asperger syndrome, but found a slight association with PDD (OR, 1.2, 95% CI, 1.0- 1.5).

Other large studies also did not find any association between tobacco exposure and ASD. In a large registry based Swedish case-control study, that included 3958 ASD cases and 38,983 controls, Lee et al. (2012) found that maternal smoking during pregnancy is not associated with increased risk of ASD after adjustments for parental education, occupation, and income.

Several additional studies (Maimburg and Vaeth, 2006; Burstyn et al., 2010) and two recently published meta-analyses by Rosen et al. (2015) and Tang et al. (2015), also reported no significant association between maternal smoking during pregnancy and ASD.

It can be summarized maternal smoking does not increase the rate of ASD in the offspring.

## Conclusions

The observed increase in the incidence of ASD in the last decades is considered to result directly from changes in definitions and better ascertainment. However, it is also possible that it reflects a real increase in the occurrence of ASD. In addition to well-defined genetic causes for ASD, examples of which we discussed in this review, the current search for prenatal environmental etiologic factors demonstrated real associations with a number of causes that may affect the developing fetal brain raising the vulnerability for ASD. Based on the findings reviewed here, we may conclude—with caution—that: (1) A possible association exists with maternal influenza in pregnancy, exposure to pesticides and insecticides, exposure to misoprostol, thalidomide, cocaine, SSRIs, or folic acid deficiency. (2) A probable association was found for maternal fever, autoimmune diseases, diabetes, preeclampsia, and exposure to heavy air pollution. (3) A definite association of ASD with maternal Rubella and CMV infections in pregnancy, maternal inflammation and immune activation, or exposure in pregnancy to VPA, and high levels of ethanol. (4) Although sometimes suggested, there seems to be sufficient evidence that there is no association with ASD for many maternal infections in pregnancy (i.e., herpes viruses, Epstein Barr virus, varicella—zoster virus, parvovirus), smoking, exposure to heavy metals or vitamin D deficiency.

Despite the increasing efforts in recent years, and support from animal studies, we still seem to be in a stage where the etiology of ASD is largely unknown and the associations described in the literature are, for many agents, inadequately proven. Hence, more research is needed to unravel environmental contribution to the genetic etiology of ASD. This type of research may ultimately lead to the establishment of possibilities for prevention.

## Author contributions

Following the request by Dr. Joshua Rosenzweig and Dr. Benjamin Gesundheit, we attach our manuscript entitled Genetic syndromes, maternal diseases and antenatal factors associated with Autism Spectrum Disorders (ASD) by EZ, WL, and OA for consideration for publication in the special topic of the Journal- Autism Spectrum Disorders (ASD)—searching for the biological basis for behavioral symptoms and new therapeutic targets.

### Conflict of interest statement

The authors declare that the research was conducted in the absence of any commercial or financial relationships that could be construed as a potential conflict of interest.
